# Responding to the call of the NHS Nightingale, but at what cost? An auto-ethnography of a volunteer frontline mental health trainer’s experiences during the COVID-19 pandemic

**DOI:** 10.1177/13591053231213478

**Published:** 2023-12-11

**Authors:** Chloe Kitto, Danielle Lamb, Jo Billings

**Affiliations:** 1 UCL, UK; 2 Royal National Orthopaedic Hospital, UK

**Keywords:** autoethnography, COVID-19, frontline, healthcare workers, impact, pandemic, qualitative, thematic analysis

## Abstract

Healthcare workers, globally, volunteered time and skills to the COVID-19 pandemic frontline response. In March 2020, the predicted high demand for extra critical care beds led to the rapid construction of the UK National Health Service (NHS) Nightingale field hospital, London. I volunteered to develop and deliver psychological preparedness training – coined ‘Psychological PPE’ – to over 2300 frontline staff over an 8-week period. Existing research has identified broad themes of the impact working on the COVID-19 frontline has on healthcare workers but does not capture in-depth accounts of individuals’ experiences. Using autoethnographic enquiry, this research explores my frontline experience at the NHS Nightingale during this time, and the personal impact this had on me. Reflexive thematic analysis explored themes of recognition and sacrifice, emotional lability and fragility, and the impact of transitions. Findings inform personal recovery, as well as future research and policy development pertaining to the sustainable recovery of our NHS people.

## Introduction

In February 2020, the UK watched as the COVID-19 virus spread rapidly across the country. The following month saw the World Health Organization declare a global pandemic: we subsequently experienced worldwide border closures, countries in lockdown, social distancing practices and personal protective equipment (PPE) becoming the norm.

Epidemiological modelling predicted the peak in UK COVID-19 cases hitting as early as April 2020, and anticipated that intensive care units across the country would be unable to cope with such unprecedented numbers (Ferguson et al., 2020). In response, NHS England planned temporary field hospitals across the UK. In London, the ExCel exhibition centre was repurposed with capacity to treat 4000 COVID-19 patients (NHS England, 2020a; BBC, 2020).

Named after the pioneering British nurse, the NHS Nightingale Hospital required thousands of health care staff to be systematically trained to deliver COVID-19-specific care. As a mental health occupational therapist, researcher and educator, I volunteered to become a ‘rapid trainer’. Over three intense days, we developed a series of innovative workshops which became the London Nightingale’s induction and training programme. We rapidly delivered this training over a 6-week period to more than 2300 staff. I advocated for and co-developed an evidence-based psychological preparedness workshop – ‘Psychological PPE’ – which became standard training for all staff (Kitto and Bakhai, 2020). My experience at the NHS Nightingale Hospital London, and the personal impact it had on me, is the focus of this research.

The impact of the pandemic on healthcare workers became a common source of discussion in the UK and globally. Commentaries and reviews of frontline experiences were published in leading journals (Chaudhry and Raza, 2020; Dubey et al., 2020; Greenberg et al., 2020; Tsamakis et al., 2020; Williamson et al., 2020), as well as newspaper interviews and blogs (BMA, 2020; Campbell, 2020; Coslett, 2020; Quinn, 2020). These report consistent themes of vulnerability, mental ill health, stress, fear, uncertainty, moral injury and burnout.

The quantitative literature on this topic consists mostly of cross-sectional surveys. A meta-review of systemic reviews of the mental health of healthcare workers found that, from a pooled sample of 3,245,768 participants, substantial proportions experienced depression (14%–37%), anxiety (16%–41%) and stress (19%–57%), with the most significant risk factors identified as being female, younger in age, and being a nurse or frontline professional (Chutiyami et al., 2022). Despite having a large pooled sample, these studies can only offer a crude snapshot of prevalence of mental ill-health, with little nuance regarding who is affected, how and why.

Early in the pandemic, a meta-synthesis of qualitative literature exploring pandemic-related frontline worker experiences identified only five COVID-19-specific studies (Billings et al., 2021b) which found wellbeing impacted by inadequate PPE, inconsistent communication from government and management, insufficient resources, reluctance in help-seeking and the benefits and burdens of peer support. A phenomenological study interviewing 20 frontline nurses from one ward found similar themes, as well as new themes such as a variety of coping styles, personal growth and simultaneous positive-negative emotions (Sun et al., 2020).

This early research shows consistent emphasis on the complex nature of frontline workers’ experiences and the subsequent impact (Billings et al., 2021a; Lamb et al., 2020). More recent work furthers these findings, identifying the socio-political contexts at the national, organisational and team levels, and the impact of these on individual healthcare workers, including a strongly felt sense of betrayal and burnout (Clarkson et al., 2023; French et al., 2022).

The large-scale quantitative and more nuanced qualitative research undertaken so far has provided useful insights. However, an important gap in the literature remains: in-depth research directly from the perspective of the frontline worker during the early phases of a pandemic, providing a deeper understanding of the recognised complexity of ‘healthcare worker impact’. While healthcare workers discussed their experiences among themselves at the time and subsequently, as noted by research that found peer support to be important, very little of this informal discussion of individualised lived experience has been captured with robust research methods (Billings et al., 2021b). Therefore, a lack of understanding remains about how best to support staff, still suffering now, from the extraordinary events of 2020–2021, and how these experiences can inform staff support in future epidemics or pandemics.

Using autoethnography, this study aimed to uncover new ideas and explanations regarding the individual experience of frontline work during the COVID-19 pandemic. Volunteering at the Nightingale during the first COVID-19 peak in March 2020 gave me the opportunity to conduct autoethnographic research, giving a scientific voice to the experiencer. My research question was: ‘*What impact did my experience as a volunteer rapid trainer at the NHS Nightingale, London, during the peak of the COVID-19 pandemic, have on me?*’

## Methods

### Ethnographic method

Literally translated as: describing or writing about (graphy) people and culture (ethno), ethnography has its origins in the study of native and indigenous peoples from the perspective of a direct or participant-observer (Ellis et al., 2010). A traditional participant-observer would immerse themselves within the day-to-day lives of the people they were studying to gain new insights about the human experience (Howell, 2018). Ethnographic research is typically inductive: aiming to first observe and describe and then to extract meaning and context to create new knowledge about culture, both for the insiders and outsiders of that culture (Ellis et al., 2010; Maso, 2001). This rich, intimate knowledge is often referred to as ‘thick description’. Coined and evolved most notably by Geertz (1973) and Denzin (1989) respectively, thick description is the descriptive *interpretation* of complex cultural situations, providing the background information necessary to understand the relevance, meaning and intention that underpins the observed social interactions (Ponterotto, 2006).

### Autoethnography

Autoethnography represents the qualitative paradigms of *constructivism* and *interpretivism*, which together suggest that the notion of reality and knowledge is socially constructed, context-specific and individually interpreted. It is because of this theoretical standpoint that autoethnographic enquiry picks up on the highly nuanced and complex nature of what it is to *experience* something (to live through an event or occurrence) and what it is to be *impacted* by something (to be effected by such an event) – both these phenomena are highly sensitive to individual context.

This research sought to employ an analytical ethnographic approach akin to Chang’s (2016), which describes autoethnography as a storytelling method, evoking the full nature of experience for the reader, but differing from autobiography in that it upholds the scientific rigour of analysis and interpretation. While some take scientific rigour to equate to removing all bias, avoidance of bias is not typically the aim of qualitative research, and in fact such methodologies – autoethnography in particular – are explicit in their acknowledgement of the role the researcher’s personal experiences, beliefs, feelings, assumptions and perceptions play in data analysis. For this reason, the role of reflexivity is especially important in autoethnography.

### Reflexivity

Reflexivity occurs throughout all parts of the research process – from data collection, analysis, interpretation and write-up – and provides the reader insight into the lens through which this research was conducted. Practicing reflexivity involves ‘self-conscious introspection guided by a desire to better understand both self and others’ within a social setting (Anderson, 2006: 382). Richards (2009) prompts researchers to ask themselves – ‘*what is interesting?*’, ‘*why is it interesting?*’ and ‘*why am I interested in that?*’. As participant-observer, reflexivity is the key to theory development, as new insights into the human experience occur in the space between self-awareness and reflexivity.

It is essential of analytical autoethnographic writing to provide the reader with an understanding of whose perspective the data and interpretation is being taken from: I am a white Australian female in my mid-30s and mental health occupational therapist of 16 years. I have worked within the NHS since 2014. Clinically, I enjoy working with people, building a deep understanding of what is meaningful to them, and problem-solving barriers which make it difficult to function day-to-day. I value working with ‘the whole person’ as it allows for an individualised and context-specific interchange. As a researcher, much like my clinical role, I am drawn to richness and context over homogeneity. This way of working flavours almost everything I do and makes up a portion of the lens through which I interact with the world. My sociocultural narrative affords me a ‘say it how it is’ tendency, a ‘service for the greater good’ attitude and a ‘rise to the challenge’ spirit.

As the virus was just making headlines, I was a happy, motivated and COVID-ignorant graduate student, taking a break from my professional occupational therapy role to complete a MSc in Mental Health Sciences Research at UCL.

*I’m so glad our life is still close to normal – [husband] and I can both work from home and [2-year old daughter] can still be with her nanny. My biggest issue is that I can’t buy flour! Not even from Amazon! (Text message to friend, 24/03/20)*

Days later, I received an NHS email requesting ‘rapid trainers’ with particular skillsets to train a large number of workforce in preparation to work in a large COVID-19 field hospital. I responded to the email, and 2 days later I was heading to the ExCel centre.

*I have officially been redeployed for Project Nightingale as a rapid trainer. . .They are hoping to take. . .500 patients on Monday; so between tomorrow, Saturday and Sunday, my job will be to teach all the staff [how] to get things up and running for Monday. . .Um. . .not going to lie - once they sent me the confirmation I was very anxious. . .not sure what that means for my study or for pay. . .not sure what that means for my level of risk, especially for [2-year old daughter]. I talked to [husband] and cried. It hit me all of a sudden. Easy to forget when you’re just at home! I just feel it’s the RIGHT thing to do. (Text message to colleague, 26/03/20)*

Friends and family would attest that I am a strong, confident and resilient person. I tend to step up to a challenge. I am a strong subscriber to the practice of *work-life balance*: what you give out, you need to get back. I find this especially true because I value giving out – in my work role, and in my other life roles as wife, mother and friend. It is likely that this spurred my decision to volunteer at the Nightingale.

The use of reflexivity and rigor allows this research to surpass autobiographical story-telling to undercover deeper understandings about the human experience.

### Design

I chose autoethnography for this research due to the depth of complexity involved in understanding *experience* and *personal impact* (Ellis et al., 2010), and the opportunity to gain new insights and develop theory through critical analysis (Anderson, 2006). On this, I followed Anderson’s (2006: 375) direction on analytical ethnography, ‘where the researcher is 1) a full member of the research setting, 2) visible as such a member in published texts, and 3) committed to developing theoretical understandings of broader social phenomena’. Subsequently, the method of ethnographic enquiry I adopted for this study was overt *participant-observation*, which informed data collection, analysis and interpretation. A participant-observer seeks to understand the human experience of their subject (in this case: myself) through *observing* their behaviour and interactions and *participating* with them in their social environment (O’Reilly, 2012).

### Setting

The Nightingale Hospital, London, was the largest of seven field hospitals across the UK, built in response to the concerning rates of COVID-19 during the first wave in 2020. The London site, built in 9 days by the military, aimed to provide overflow capacity for patients to receive critical care away from general hospitals. Simultaneous to the building of infrastructure, staffing teams were rapidly developing procedures, protocols, risk assessments and training programmes to support the mass induction of workforce that was required to staff the field hospital. As Wellbeing Lead, I co-developed the ‘Psychological PPE’ training programme, recruited trainers from across the country, and successfully delivered this novel training to over 2300 newly inducted staff. The success of the NHS Nightingale hospitals has been widely debated; but at that time, in March 2020, despite fear and epic levels of intensity, thousands of volunteers stepped forward to be part of the ‘hopeful solution’ (Anandaciva, 2021). This paper captures this experience first-hand.

### Data collection

A participant-observer typically captures data by writing detailed fieldnotes documenting daily experiences and new insights. In this study, data collection occurred over 12-weeks from late March to mid-June, 2020. This time period included the 8 weeks I was working as a rapid trainer across the two London Nightingale sites – the ExCel Centre and the O2 Arena – and the following 4 weeks post-Nightingale when I was home-based.

Wolcott (2009) urges qualitative researchers to explicitly detail how participant-observation occurred. My first few notes were brief and lacking detail, but as my familiarity with this method grew, I wrote using the following structure: through the lens of ‘observer’ I asked myself – ‘*what happened today?*’ and recorded day-to-day events, routines, interactions, environments, milestones and changes in detail. Then, adopting a ‘participant’ lens, I would ask myself – ‘*what did I personally experience today?*’, and recorded emotions, thoughts, *relationships* and personal struggles. After taking two distinct columns of notes, I would then reflect on the meaning and interpretation of the story told. Auto-enquiry, like this, involves discovering new and interesting facets of your own interaction within a social context; and these, as they emerged, were documented.

I habitually spent my 45-minute commute home reflecting on the day and, using the prompt questions above, entered fieldnotes into the ‘Notes’ app on my phone. Due to the intensity of activity and time pressures at the Nightingale, it was impractical to walk around the hospital with a notepad. I occasionally had the opportunity to enter a fieldnote during the day, for example, waiting for a meeting to start or taking a bathroom break, or more notably, whilst travelling between the two Nightingale sites on the free-to-NHS-staff Emirates cable cars.

**Figure 1.**
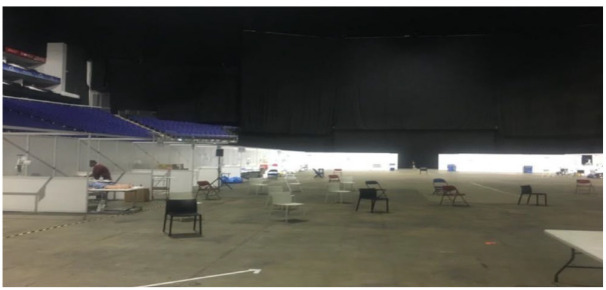
Clinical simulation teaching area, O2 arena (20/04/20).

As well as fieldnotes, I included Nightingale-related data from voice messages sent to friends throughout the data collection timeframe, which added emotional depth to participant-observation. Items of memorabilia, including lunch tickets, security wristbands and photos of the environment, were also collected as a deliberate strategy to sharpen my recall during the analysis and write-up stages (*see* Figure 1).

My fieldnotes ended when the NHS Nightingale ceased operation. My reflective notes continued as I ‘came down’ from the frontline experience over a period of several months, and ceased due to the time limits of this study.

### Data management and analysis

I chose to deliberately distance myself from the data for a 2-week period before I transcribed the voice message data verbatim and added it to my written fieldnotes. This distance aimed to create space from my role as data collector (participant) and support the transition into data analyst (observer). I input all data into NVivo (Pro V.12) and reviewed it as a chronological narrative timeline. Reflexive thematic analysis, as outlined by Braun and Clarke (2021), was chosen because of its shared epistemological and ontological orientation with ethnography. It upholds the subjective experience as an analytical and interpretive resource by which to produce insight and theory. The analytical process began through total engagement with the data. Broad coding was used to identify patterns within the data of interesting sociocultural experiences and behaviours. Similar codes were then clustered and thematic development occurred inductively throughout a process of auto-enquiry and reflexive dialogue. Memos (in NVivo) were used to document the reflexive process, capturing the maturation of themes and their interpretation. The final themes were chosen as per their significance to the research question. Unlike other analysis which utilises multiple researchers to reduce bias, autoethnographic analysis is a solo pursuit, purposely digging deeper into the researcher’s own analysis of their own observations.

### Ethics

Ethical approval granted by UCL Research Ethics Committee [Project ID: 18441.001]. No names or identifiable descriptions of others are included. The risk to researcher was considered and supported through self-monitoring and supervision. The disclosure of experiences and vulnerabilities can leave the researcher open to critique from the public, potentially impacting reputation and wellbeing. For this reason, information shared in this study has been carefully considered. That said, few sensitive details were omitted in favour of richness and authenticity.

## Results

**Figure 2.**
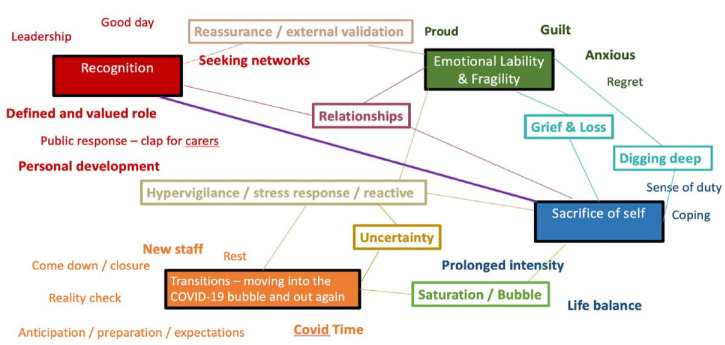
Infographic of thematic analysis, showing key themes and their interactions.

The themes explored in this study are the most meaningful to me as participant-observer. Figure 2 shows an infographic depiction of the key themes explored in the thematic analysis. It might read messy – it’s intended to. It represents the complex and complicated inter-relational nature of the presenting themes. The connecting lines represent mutual interactions between themes, with bolder lines and text emphasising stronger associations.

Within the interpretation, the themes of *recognition* and *sacrifice of self* were combined into one and critiqued as one dynamic interaction. Subsequently, three key themes are discussed – (1) *Recognition versus self-sacrifice*; (2) *Emotional lability and fragility*; (3) *Transitions*. Inter-related themes throughout are written in *italics.*

### Theme 1: Tipping the balance between recognition and sacrifice of self

The theme of *recognition*, presented here as a complex concept, includes the idea of reward, *reassurance and external validation*. In my normal work setting, as an occupational therapist in the NHS, recognition is low-key and comes from patient gratitude, team working and subconscious self-appraisal. I did not volunteer at the Nightingale for money, praise or glory, and I do not typically seek out overt recognition.

As this theme grew within the data, it challenged me to think about *why* recognition was so important to me now. I felt confronted by its prominence, trying to reconcile it with myself – surely I am not this kind of ‘needy, praise-seeking’ person?! That’s not like me. It made me feel embarrassed. If I return to the above reflections on recognition within my usual work role and compare it to my Nightingale experience, there is a suggestion of a recognition deficit. For instance, I did not have the opportunity for patient gratitude. I did have the reward of teamwork to an extent; however, as a team leader, I was less *within* the team and separated somewhat from peer comradery. What is most interesting was my ability to achieve recognition via self-appraisal was tangibly lacking. Instead, I was heightened and dependent on recognition (in the form of *reassurance* or *validation*) from others. Extrinsic rather than intrinsic. There was a pervasive need to be justified. A need for the sacrifice incurred by such a demanding and *saturating role* to be for a reason. I needed to be reassured that I was doing the right thing. I continue to feel this even now, as I write this article months later.

*I felt good again being part of the inner crew. . .important and utilised and valued. I’m glad I have the capacity to stay on with the Nightingale for however long. I like hearing from others that my role here is really worthwhile. And I feel proud to be liaising with big-wigs regarding the wider wellbeing of the hospital. I’m trusting that my work here and the sacrifice of family and uni, etc, will pay off somehow. There has to be something good to come from it, right? (Field note, 11/04/20)*

**Figure 3.**
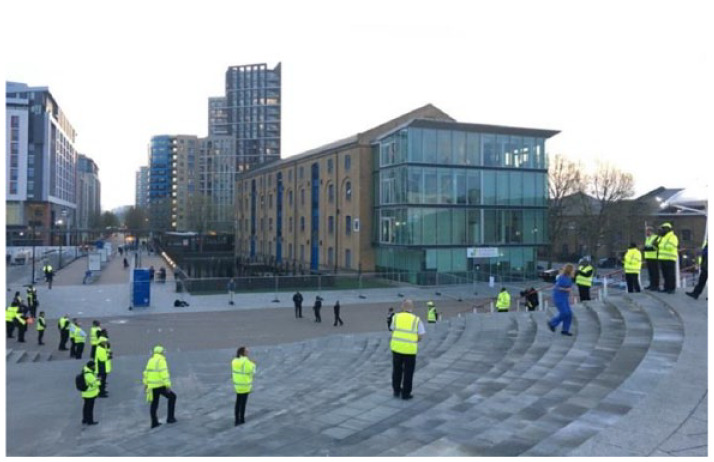
NHS Nightingale staff are clapped as they arrive for their evening shift (09/04/20).

Remarkably, extrinsic recognition did exist in the form of a nation-wide, thunderous applause every Thursday night for 10 weeks with the ‘*clap for carers*’ campaign (*see* Figure 3).

*I am just walking out of the ExCel centre to jump on the DLR and am floored by the clapping, vuvuzelas, pot drumming, whistling, honking and fireworks from surrounding apartments. I hadn’t even realised that it’s Thursday today, let alone 8pm! I took a picture because it was my first moment of overwhelm at the job we’re trying to do. I wanted to remember the moment, I guess. (Field note, 02/04/20)*

Later in the same note, I had boarded the train and wrote. . .

*The overhead announcement just said - “Docklands Light Railway would like to thank NHS staff for their ongoing commitment”. It’s so crazy how everyone is really getting behind the NHS, from balconies in high-rise apartments to public transport announcements! I guess as everyone sits at home they are depending on us to save the day. We are being called ‘heroes’ now, but actually a lot of the workforce I’ve trained really hate that. It’s pressure. Especially as we’re about to scale up and need to dig deeper! (Field note, 03/04/20)*

The positive recognition from the public was quickly reframed into pressure to keep giving more of myself. This excerpt was taken from an early fieldnote and it demonstrates the beginning of an association between *recognition* and *sacrifice of self* within this new sociocultural context.

The *sacrifice of self* theme within this research is enormous and debilitating, impacting all areas of life which hold value. When social restrictions commenced, like many, I grieved for the loss of social participation – daily habits of grabbing a coffee and the practice of my usual self-care activities, like going to the gym and laughing with friends.

On top of this, however, because of my choice to volunteer at the Nightingale, I lost my opportunity to learn and succeed in my Master’s programme, and the financial sacrifice we made for this; I lost precious time with my 2-year-old daughter, missing bedtimes and milestones, and losing the basic mental capacity to be a present care-provider; I lost the ability to be a good friend and wife, even losing a friendship because of this, and my marriage suffered immensely; I lost the capacity to self-care, gaining substantial weight with poor eating and drinking habits, and allowing an ‘I don’t give a shit’ attitude to become a mental weed. And I lost my mental health. The healthcare professional in me cringes at this admission. I am the one who helps people, not the one who needs help!

My mind and emotions are not great. I don’t have a common sense and I don’t have reason and rationale like I usually do. I’m starting not to trust my judgment. . .*cries*. . . I just feel like all the things I value most in life I just haven’t invested in them. I feel disappointed in myself and I think, like, why did I even go to the Nightingale in the first place? It wasn’t worth this. . .It just seems like it’s affected every single part of my life. Right now I feel it wasn’t worth it. . .and that’s a really difficult thing to be thinking now as I try to come down from it all. I’m waking up to a really shit reality. (Voice note to friend, 31/05/20)

Analysing the interaction between *recognition* and *sacrifice of self* has allowed me insight into the mechanisms behind my out-of-character relationship with recognition, and further, into the overall impact of this experience on me. It describes an adaptation that took place amidst a new and demanding sociocultural setting: if sacrifice here speaks to all of the ways I *gave out from myself* as a volunteer at the Nightingale, then recognition-seeking became a new tool to (try to) *give back to myself* within this context.

The commonly used ‘empty cup’ analogy comes to mind, often used in mental health self-care dialogues: ‘You can’t pour from an empty cup’. My cup was beyond empty; it was (is) shattered. My role at the Nightingale did not offer my usual sources of recognition and reward, as described above, which may have filled my cup. Nor did the experience allow for self-care. Arguably, the pandemic hindered the usual self-care of many people, unable to ‘fill their cups’ in their usual way. But the relentless urgency, the constant outward focus, always ready to respond – this prevented any sensible process of adaptation. So I sought *recognition*, and looked to others for *reassurance* and *external validation*. My inner monologue saying – ‘I’m here, and this is important, but my also, I’m not really okay, and my life is really falling apart in big and small ways, which I can’t really pay attention to right now, but I’m doing the right thing. . .right?’.

Exploring this thematic interaction further, I observed that my ‘*good days*’ in the fieldnotes commonly included themes of *recognition* and validation, displacing the feelings of sacrifice. My ‘bad days’ would commonly describe *sacrifice of self* content. I attributed the positive feedback from the trained workforce, and how this fed my sense of reward, to having a ‘good day’.

*It was really satisfying to lead four Psychological PPE groups today. It was more than I have done in a while as I’ve been busy with team management, recruitment and finalising the lesson plan. It was really nice and right in my comfort zone. More than anything else the reward I get is the nonverbal and verbal engagement from the workforce when they invest in their mental health and the mental health of their colleagues. It feels good to actually do what I came here to do! (Field note, 20/04/20)*

Other ‘good day’ content included: *networking* with influential people where I was seen and known, having a *defined and validated role* within the wider Nightingale project, or being able to promote the wellbeing work to a new audience.

*I felt very fulfilled today. I felt like a good leader and I got good feedback from my team on my facilitation. They said I was passionate, enthusiastic, and that I know my stuff. It’s nice to be validated! I enjoyed attending the hospital meetings today too because I felt influential and valued. It means a lot to me to be included in these hospital-wide conversations about staff wellbeing. (Field note, 27/04/20)*

Analysing the interaction between *recognition* and *sacrifice of self*, I was interested to see if a healthy balance was achieved, particularly considering this process of adaptation to a new context. What I found was that the recognition (reassurance/reward/validation) I received, despite needing it at the time, served to perpetuate my *sense of duty*. In turn, it pushed me to *dig deeper* into the sacrifice by inadvertently disallowing me to stop, to take a break, to walk away. There also appeared to be a cyclic nature to this interaction – *sacrifice of self* leads to the pursuit *recognition* which perpetuates the *sense of duty* which forces me to continue the sacrifice of self, and so on. This recognition-based (mal)adaptation that I observed in my fieldnotes, which aimed to refill my cup, served to, in fact, deepen the cracks.

### Theme 2: Emotional lability and fragility

Analysing the richness of my emotional experience at the Nightingale would be, on its own, enough to fill a research article. This theme was prominent throughout my fieldnotes and one which I struggle to engage with at length. I am normally very comfortable with emotion and can moderate my emotional expression as needed. I was, therefore, surprised to observe such a pronounced lability in my emotions, even from very early on.

*First day of psychological PPE as a skills station. . .The ExCel centre is incredibly spacious and I feel for these people walking from one end to the other, going in and out of identical rooms trying to figure out if they are in the right room. The timetable did not work today. We were constantly on whatsapp reacting to last minute room/time changes. . .The feeling of moving from chaos! organising! leading! problem solving! to a slowed-down “welcome to psychological PPE, how’s everyone feeling?” is taxing. . .I feel so good at how impressed people were to see wellbeing content included!! We’ve been pushing hard for it to make the cut. It was concerning to hear that it was not usual for health care professionals to think about self-care. People are already anxious. We have been encouraged to “meet uncertainty with uncertainty” . . .but I just feel like when a person sits in front of you and says they feel anxious. . .I want to give them support. . .[Another] thing that happened today. . .I saw some of my work colleagues! The extrovert in me was satiated at last! Face to face contact with friends! I think my spirits are lifted. 0900-2200 workday. (Field note, 01/04/20)*

*Emotional lability* describes strongly expressed emotions changing quickly over a period of time. At its most severe, it is a disabling symptom of some psychiatric and neurological conditions, but here, it is used to capture the intense throes of emotion described throughout my fieldnotes. Insignificant successes would result in an exaggerated fanfare:

*As I was exploring the O2 wings, trying to establish which dressing room would make the best wellbeing room, [I was] let in on a secret: a speakeasy room, known as “The Fab Room”! As [I] opened the door, I saw a very small, very kitsch front room. It had floral wallpaper and lots of hanging photo frames. The back wall was a wardrobe. . .but opening the wardrobe door led into a lava lounge nightclub room! . . ..which then, via the bookshelf on its back wall, opened up into an arcade games room! I was adamant. . .to be the person to show everyone the secret room. I spent the next hour giddy with excitement, thoroughly enjoying letting people in on the speak easy secret one-by-one, and making them swear they wouldn’t tell so I could be the one to show and tell! (Field note, 11/04/20)*

A perceived lack of communication and trust within my team resulted in accusations of betrayal and blind-siding hurt:

*I feel very emotionally charged up this evening. Obsessed and saturated to be honest! Very disrespected, I feel cheated and wronged. There’s an injustice here which makes me more angry since these are my teammates. The reason for my poor sleep and the fuel poured on this emotional flame at the moment was just now seeing a twitter chat based on* [the issue at hand] *which none of us were told about or invited to join. There have been a number of opportunities to share the fact that an academic Twitter chat was happening, and yet. . .none of the team were invited to join. (Field note, 16/04/20)*

**Figure 4.**
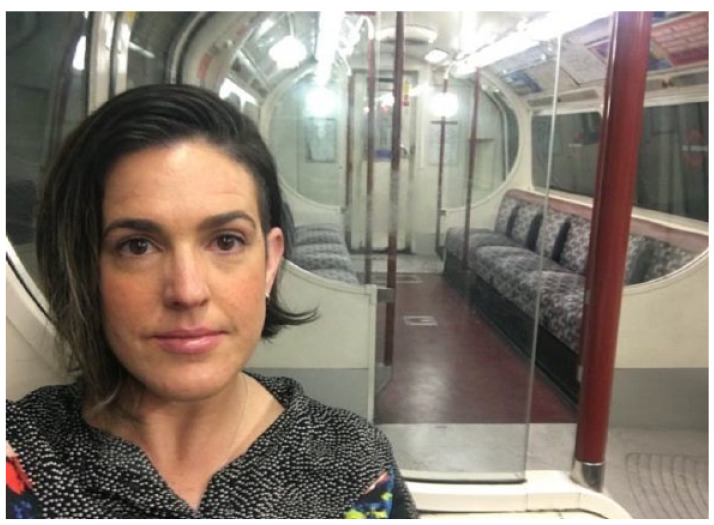
Emotional moment travelling home on the empty Bakerloo line after the announcement the Nightingale will close within the week (07/05/20).

Unremarkable events seemed to induce a heightened response, characterised by exaggerated emotion. I observed within the data that often surrounding these labile episodes were significant stressors and/or big change moments (*transitions*) (*see* Figure 4).

The phrase – ‘The train is already moving, we’re just throwing down the tracks as we go!’ was often used by our leadership team at the Nightingale. Imagine it for a second. It’s a highly effective analogy for representing the feelings I had of urgency, hypervigilance and immense responsibility under high pressure. The critical analysis process of this research has shown me that my Nightingale experience was a constant and prolonged stress response. This may seem over-simple, but the stress response is a whole-body state – physical, cognitive, emotional, biological – driven by the perception of a threat. It creates a human ready to respond. It can be switched on (triggered by a threat) and it needs to be switched off (either by removing the threat or by activating the calming side of the nervous system). Emotional regulation, at this time, was unavailable to me. The train analogy above paints a picture of threat-after-threat-after-threat. My emotions were at the mercy of the next stress response, whilst simultaneously recovering from the last one.

*Emotional fragility* on the other hand describes the insufficient resources, or *coping skills*, required to manage strong emotions. I had never experienced this before. That is not to say I’ve never had periods of struggle in life – of course I have – but I have always found ways to cope. Just writing that last sentence is a confronting admission – why did I not find ways to cope this time?

The recurrent stress responses heightened my emotional state whilst reducing my introspection. I could not see that I was not coping well. My focus was outward – tasked with looking out for the wellbeing of others within a context marked by hypervigilance. It disempowered me to consciously consider the coping that I needed. Interestingly, this may be one mechanism behind why I sought recognition and external reassurance to fill my cup, and to cope. A maladaptation based on what was accessible to me in my limited external line of focus.

### Theme 3: The significance of transitions

It would be accurate to say that the entire Nightingale experience was made up of transitions, big and small, but all significant because of the unpredictability of the Nightingale’s sociocultural context. A *transition* was as routine as going home every day and trying to switch off, and as daunting as being relocated from the ExCel centre to the O2 Arena in 1 day to scale-up training delivery from 150 to 1000+ workforce a day. I found switching off the hardest – I didn’t switch off. How can one switch off when the train is still motoring forward?!

*I had an argument with [my husband] about how immersed and distracted I am with the Nightingale and how he wants me to be more present at home, especially with [2-year old daughter]. I’m ALWAYS on my phone because Nightingale whatsapp groups are pinging 24/7 and I have bad FOMO. I know everyone is still there working crazy. I’m just [at home] waiting to go back. (Field note, 13/04/20)*

Throughout my fieldnotes, I observed that *transitions* were challenging – a source of uncertainty, anticipation, expectation and emotional lability and fragility. I discovered that *transitions* had a significant indirect association with the other key themes by being a trigger of the stress response previously described. In this context, it was a source of threat.

Exploring *transitions* as threat brings me back to the train analogy. As we were throwing down the tracks, a change in direction (transition) meant rapid adaptation under pressure. Typically, this would refresh the drive to *dig deeper*, especially when the transition was unpredicted or not meeting expectations.

*More new faces in the faculty today. No time or space for formal introductions or “here’s our story so far”. I was expecting just two new recruits to the wellness team today but we have five and no spare trainers to rapidly train them up. I’ve had to book in a mock training session which I’ll run over lunchtime so they can at least have a go at delivering a session. . .Every time we get new staff it’s like we take two steps back and just loose our momentum. (Field note, 06/04/20)*

Probably the most impactful transition was the ‘*comedown*’ from the Nightingale, which was put on stand-by as a field hospital in May, 2020. Once the Nightingale *bubble* had burst, I was acutely impacted by the *grief and loss* and sacrifice detailed in the first theme. The bubble had been a protective feature, blinding me from the reality happening outside of the Nightingale, and during this comedown period, I was hit with it all. Interestingly, because I was still in coping mode – digging deep, not able to be introspective – I was not aware I was struggling. Additionally, my new coping adaptation of recognition and external reassurance was incompatible with my normal life, where people were waiting for me start giving back to them.

*I think I have a very good ability to be strong and resilient when I need to be and take a lot of things on and just be the one that does the things. Maybe it’s because I am getting older or because I have a child, but the fallout of being strong is. . .I don’t even know. . .it’s surprised me that I haven’t been able to bounce back. I haven’t been able to be productive, to think clearly, to have motivation. Instead, I’ve had actual fatigue and anxiety and just wanting to block it all out. Just check out. I’ve had an emotional couple of weeks coming down from the Nightingale. I’m just trying to get back in touch with myself. (Voice note, 31/05/20)*

**Figure 5.**
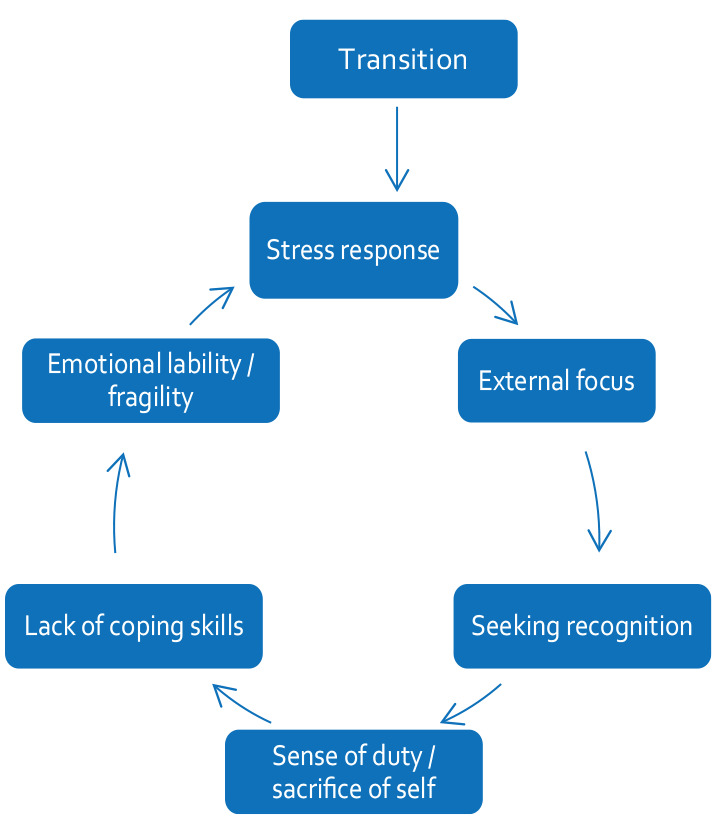
Cycle of impact – the complex interplay of the key themes within this research.

Overall, the way in which these themes interacted was complex. Figure 5 shows this complex interplay of themes as a *cycle of impact*.

## Discussion

In this research I sought to explore my experience as a volunteer rapid trainer at the NHS Nightingale Hospital, London, at the first peak of the COVID-19 pandemic, for the impact it had on me.

Roy and Uekusa (2020) emphasise the ‘once-in-a-lifetime opportunity’ found within this global pandemic to conduct in-depth qualitative research on people’s experiences. They specifically highlight the methodological and ethical advantages of using autoethnography as it is not limited by social restrictions and avoids the risk of exploiting people during challenging circumstances. There is a need for a methodology like autoethnography which has the critical precision to produce rich, experiential data from real-time data collection and self-reflexivity (Roy and Uekusa, 2020). This is in contrast to much of the current literature in this field which relies on retrospective data collection. The timepoint at which these data were collected is of particular note, given the lack of in-depth evidence about the experiences of frontline workers at the start of the pandemic.

The reflexive thematic analysis used in this study was conducted in accordance with established guidelines (Braun and Clarke, 2021) to allow for an organic process of theme and theory development. The result was three themes: (1) *Recognition versus self-sacrifice*, (2) *Emotional lability and fragility* and (3) *Transitions*. The themes on their own offer intimate insights into my Nightingale experience; however, interpreting the themes inter-relationally – as they influence each other – gave meaning and mechanism to the personal *impact* this experience had on me.

The theme of *transitions* was a constant thread across the data. This ever-changing landscape primed me to sustain a stress response characterised by hypervigilance and responsiveness. This, in turn, kept my focus on the external environment, ready for whatever came next. The absence of introspection inhibited my self-evaluation and ability to adapt effectively to the new environment. I, therefore, sought new coping skills from within my extrinsic focus, which was a key influencing factor on my *recognition*-seeking behaviour. The recognition and validation I received, however, drove my *sense of duty* and *sacrifice of self*, which I sustained without an effective way of coping, leading to a sense of *digging deeper* and allowing myself to exist in stress-response mode.

The *comedown* from my time at the NHS Nightingale lasted several months, certainly longer than the period of data collection. The *cycle of impact* has lasted years. This intense coping adaptation changed my behaviour, my emotional reactivity, my ability to introspect, my self-confidence and it didn’t revert back to normal once my frontline experience ended. That sense of urgency and reactiveness remained, triggered by everyday transitions. I recall a recent experience where my daughter and I needed to catch a bus. Upon seeing it coming down the road from my front door, I yanked my daughter, half-dressed, out into the street, yelling at her to hurry up, to not worry about her coat – ‘We can put it on in the bus!! We must run, babe!!’. I ran out across the busy road, daughter being pulled along behind me, stopping traffic and frantically waving at the bus to wait for us. . .

Moment of transition, stress response, outward focus, lack of coping skills, emotional lability – it’s all there in that example. Another bus would have come in 7 minutes. The cycle of impact – my impact – has remained.

Regarding ‘impact’ on healthcare workers, the early literature identified broad themes – depression, anxiety and stress (Chutiyami et al., 2022) and ‘concern for others’ (Billings et al., 2021a, 2021b) – with recent literature using richer analyses, specifying themes of moral injury (Hegarty et al., 2022), ‘toxic stoicism’ (Clarkson et al., 2023), burnout, and betrayal (French et al., 2022). This study can draw similarities to these standalone themes but adds depth, with findings suggesting the impact on healthcare workers is highly complex and full of personal nuance (Billings et al., 2021b; Lamb et al., 2020). It suggests that within one person’s experience there may be many dominant themes having an impact, and further, as found in this study, there may be a deeper level of impact caused by the dynamic interplay of the themes (see Figure 5).

In their review of stress and health literature, Segerstrom and O’Connor (2012) emphasise the importance of framing stress as a highly nuanced experience. Factors such as how one perceives their environment, coping as an interaction between person and stress and how this interaction changes within one person’s lifespan describes a complex and dynamic understanding of stress, which is echoed heavily in our research. I have always been a highly resilient person with healthy coping strategies, but evidently, the specific factors (personal, environmental, behavioural, temporal) which interacted to make up my experience of ‘stress’ as a frontline healthcare worker impacted my usual ability to cope and maintain my wellbeing. Interestingly, Segerstrom and O’Connor (2012) promote the use of real-time data collection to help capture this ‘nuanced narrative of stress’ as it presents within a particular person, at a particular time, within a particular environment.

Importantly, there are themes within qualitative literature which are in direct contrast to my ‘impact’. For example, Aughterson et al. (2021) conducted interviews with 25 frontline workers in the UK exploring the psychosocial impacts of COVID-19 work. Participants exhibited significant psychological resilience and this was linked to ‘proactive coping’ using well-developed coping strategies. They also described a relatively healthy comedown process, where they were able to reflect, slow down and take stock of what matters to them in life.

It’s at this point I find myself asking: *why did this experience have the impact on me that it did?* What were the factors that led me into the *cycle of impact*? Why was my experience different to the participants in the Aughterson et al. (2021) study? I also approached my COVID-19 work with well-developed coping strategies and high levels of resilience. These things which have protected me from burnout and mental ill health throughout my career did not serve to protect me this time – *why?*

Drawing from Segerstrom and O’Connor (2012), perhaps the mechanism behind my experiences of impact from frontline work resides in my ‘nuanced narrative of stress’ – all the micro-events, compounding over time, influenced by what was going on for me, at that particular time, hour by hour, in that particular environment. Highly individualised. Highly contextualised.

This chimes with my strong tendency towards a whole-person frame-of-reference, as described earlier in my statement of reflexivity. Occupational Therapists are trained to develop very individualised and context-specific treatment plans, because we know recovery works when individuals are able to meaningfully participate in therapy which considers all functions of their daily life. The analytic process within this study has reinforced the importance of framing the *impact* that COVID-19 frontline work had on me as a whole-person experience, and points towards the need for support in a highly individualised way, according to a person’s ‘nuanced narrative of stress’.

The findings of this study indicate a coping adaptation, occurring in response to a new sociocultural environment marked with uncertainty, constant urgency and stress, the absence of usual coping practices and the inopportunity to develop (healthy) alternatives. Furthermore, the adaptation intended to bolster my coping instead acted to reinforce my self-sacrificing behaviours. My awareness of this maladaptation process (and its impact on me) has been highlighted only as a result of this autoethnography and the reflexivity component of the analysis. I highlight this only to wonder – were it not for this study, would I still be stuck in a cycle of maladaptive coping?

### Strengths and limitations

This autoethnography addresses a clear research gap in the literature by providing a first-hand, real-time ‘experiencer’ perspective. It provides rich, descriptive insights into the personal impact of frontline work, and the deeper mechanisms which influence behaviour, social interaction and the complexity of the human experience within the Nightingale context. This methodology maximised the depth of enquiry to address the research question and was conducted in line with Le Roux’s (2017) guidelines for scientifically rigorous autoethnography.

Nevertheless, this study is also subject to limitations. Leaders in autoethnography (O’Reilly, 2012) and reflexive thematic analysis (Braun and Clarke, 2021) suggest that good analysis depends on the expertise of the researcher. Subsequently, it is important to declare myself as a novice in both these skillsets and the impact this may have had on the quality of my research. Secondly, the data collection was limited by fixed time parameters surrounding the research study. Remaining in the ethnographic experience for longer may have added even more depth to the research question. Lastly, the nature of this methodology may provide more questions than answers. It does not provide evidence for the effectiveness of particular avenues of support for healthcare workers, but offers new areas of inquiry which can be used to inform the development of targeted support.

### Implications

Chang (2016) states that ‘auto-ethnographers should not only tell evocative personal stories for reader reactions but also make serious efforts to speak to what their personal issues should mean to the broader health research community’. This research highlights important implications at an individual, research and policy level.

#### Individual

As a self-enquiry process, this research has allowed me time and space to process my experience that I would not otherwise have had. I have experienced exceptional *personal and professional growth* because of this research which will directly impact how I prepare myself for future crisis-driven work. As an occupational therapist and educator, the insights I’ve gained from this study have enhanced my clinical and academic provision to others. I have been able to critique myself as a leader allowing me significant maturation within this role.

This research process has highlighted the beneficial role of supported reflection in order to understand my own ‘nuanced narrative of stress’ and facilitate adaptation, growth and recovery. Further, the recovery to be gained through the reflective process may be enhanced by taking a constructive approach and ensuring structure and momentum are present. My ‘time and space’ for reflection to process my frontline experience was given to me by the scientific research process (structure), which was self-paced but supported by supervision (momentum), and which, by its nature, upheld a practical and non-judgemental focus (constructive approach).

It is a probable assumption that, similar to me, other healthcare workers have been negatively impacted by their own ‘nuanced narratives of stress’. According to the 2019 NHS Staff Survey, high levels of stress and burnout were already present among healthcare professionals prior to the COVID-19 pandemic, with factors such as reduced autonomy, excessive workload, unpaid overtime, compromised safety culture and inequality contributing to healthcare staff being 50% more likely to experience work stress than other industries (West, 2020). The various effects of prolonged stress on physical and mental wellbeing and behaviour are well described in the literature, however, it is often over-simplified.

To value the recovery of healthcare workers is to value the complex and very un-simple nature of the impact that prolonged stress has had on the individual lives of healthcare workers, before, during and after the pandemic. The benefit of quality time and supported space to process complex narratives of stress, for the purpose of sustainable recovery, is likely to be beneficial to those who responded to the pandemic.

#### Research

Current literature suggests broad areas of impact on frontline staff. This study should be used as a catalyst to conduct further qualitative research, digging into the contextual factors which influence *experience* and *impact*, such as behavioural and coping adaptions, individual narratives of stress and the impact of stress-inducing, rapid transitions. Furthermore, exploration of the potential mechanisms highlighted in this study, which underpin *impact*, would significantly contribute to developing support strategies for frontline workers, as recommended by Billings et al. (2021b). For example, sense of duty and its influence on self-sacrificing behaviour, or, supported introspection as a protective wellbeing skill.

The unique context of this pandemic has shown that healthcare workers may have relied more heavily on external sources of support with varying outcomes. Our finding explored themes of external validation and recognition-seeking, and other studies have explored the positives and negatives of social and peer support (Billings et al., 2021a, 2021b) and the impact of senior leadership (French et al., 2022). Therefore, there are significant insights to be gained by researching the nuances of leadership styles and peer support culture and their mechanisms of impact on the experience of healthcare workers.

It is worth considering in future work how best to use evidence from ethnographic research, whether (and if so, how) such evidence converges with other types of qualitative (and quantitative) work, and what a methodologically-inclusive research landscape can add to organisational and policy decisions regarding the wellbeing of healthcare workers. It has been argued that the term ‘triangulation’ insufficiently captures what is most important about incorporating evidence from different methodologies (Fetters and Molina-Azorin, 2017) and that convergence, complementarity and divergence may provide more useful lenses through which to combine evidence (Morgan, 2019).

Carrying out this research created an awareness of maladaptation processes, specifically by the note-taking and reflexivity required by the autoethnographic methodology, and suggests that future research could usefully focus on the benefits of expressive writing and reflexive approaches. There is some evidence from a randomised controlled trial carried out during the pandemic that expressive writing was beneficial to healthcare workers (Procaccia et al., 2021), and this bears consideration for future intervention development.

#### Policy and organisational response

There is an ongoing agenda to address the wellbeing, staff experience and sustainability of the NHS workforce. The ‘NHS People Plan (2020/21)’ directly acknowledges the impact of the pandemic and advocates for staff to address their own health and wellbeing (NHS England, 2020b). Now, in 2023, there appears to be a tangible absence of opportunity – time, space, quality support – to enable staff to address their own health and wellbeing, with the daily burden of unprecedented waiting lists, prolific vacancies across services, poor recruitment and the compounding effects of high levels of staff stress and burnout.

Findings from this study could significantly contribute to understanding the complexity of workforce wellbeing and inform the strategies and resources required to help staff effectively recover. The highly individualised nature of impact indicates the highly individualised nature of recovery. This may present as different people needing different things at different times, as determined by the individual.

It is a systemic challenge to invest in the individualised recovery of healthcare staff in this way, especially within the current socio-political climate. The ‘NHS People Plan (2020/21)’ recommends every member of the NHS have a personalised wellbeing plan shared within a wellbeing conversation with their line manager. This is very promising, and chimes with the findings of this study; however, it risks being a tick-box activity without significant service planning, organisational strategy and resourcing to allow it to have real and meaningful impact.

In addition to a wellbeing plan, attention must be given to what quality, individualised support is on offer. Much of the support commissioned for frontline staff currently falls within the mild-moderate psychological domain. For many, this will be incompatible with their own ‘nuanced narrative of stress’, and may not be where they feel comfortable to first seek help. If we acknowledge that impact effects the whole-person, then we must create and equally promote targeted support across all domains of life. This allows an individualised wellbeing plan to manifest into individualised wellbeing action.

## Supplemental Material

sj-docx-1-hpq-10.1177_13591053231213478 – Supplemental material for Responding to the call of the NHS Nightingale, but at what cost? An auto-ethnography of a volunteer frontline mental health trainer’s experiences during the COVID-19 pandemic Supplemental material, sj-docx-1-hpq-10.1177_13591053231213478 for Responding to the call of the NHS Nightingale, but at what cost? An auto-ethnography of a volunteer frontline mental health trainer’s experiences during the COVID-19 pandemic by Chloe Kitto, Danielle Lamb and Jo Billings in Journal of Health Psychology

sj-docx-2-hpq-10.1177_13591053231213478 – Supplemental material for Responding to the call of the NHS Nightingale, but at what cost? An auto-ethnography of a volunteer frontline mental health trainer’s experiences during the COVID-19 pandemic Supplemental material, sj-docx-2-hpq-10.1177_13591053231213478 for Responding to the call of the NHS Nightingale, but at what cost? An auto-ethnography of a volunteer frontline mental health trainer’s experiences during the COVID-19 pandemic by Chloe Kitto, Danielle Lamb and Jo Billings in Journal of Health Psychology

sj-docx-3-hpq-10.1177_13591053231213478 – Supplemental material for Responding to the call of the NHS Nightingale, but at what cost? An auto-ethnography of a volunteer frontline mental health trainer’s experiences during the COVID-19 pandemic Supplemental material, sj-docx-3-hpq-10.1177_13591053231213478 for Responding to the call of the NHS Nightingale, but at what cost? An auto-ethnography of a volunteer frontline mental health trainer’s experiences during the COVID-19 pandemic by Chloe Kitto, Danielle Lamb and Jo Billings in Journal of Health Psychology

sj-docx-4-hpq-10.1177_13591053231213478 – Supplemental material for Responding to the call of the NHS Nightingale, but at what cost? An auto-ethnography of a volunteer frontline mental health trainer’s experiences during the COVID-19 pandemic Supplemental material, sj-docx-4-hpq-10.1177_13591053231213478 for Responding to the call of the NHS Nightingale, but at what cost? An auto-ethnography of a volunteer frontline mental health trainer’s experiences during the COVID-19 pandemic by Chloe Kitto, Danielle Lamb and Jo Billings in Journal of Health Psychology
