# Supplementary material for: Responding to the call of the NHS Nightingale, but at what cost? An auto-ethnography of a volunteer frontline mental health trainer’s experiences during the COVID-19 pandemic
Source: J Health Psychol. 2023 Dec 11;29(6):534–51. doi: 10.1177/13591053231213478 (PMC11075404; doi:10.1177/13591053231213478)
Supplement: sj-docx-1-hpq-10.1177_13591053231213478 – Supplemental material for Responding to the call of the NHS Nightingale, but at what cost? An auto-ethnography of a volunteer frontline mental health trainer’s experiences during the COVID-19 pandemic [file sj-docx-1-hpq-10.1177_13591053231213478.docx]

Data set

Given the type data used in this analysis, that is, personal notes, memos, and texts, for ethical reasons the raw data is not available.

While we support the principles of open data and the goals of enabling replication of analyses, the nature of an auto-ethnography means that replication is not only not possible, but is not desirable or appropriate. An auto-ethnographic analysis is, by definition, not replicable: a third party would not approach or analyse the data in the way the first author did, precisely because they are not the first author. The **self**-reflection required by auto-ethnographic methods is only possible by the person who shares and analyses their unique experiences. Any attempt by a third party to replicate such an analysis is impossible, as the third party cannot directly access the first author’s perspective in the way an auto-ethnography requires.
