# Supplementary material for: Responding to the call of the NHS Nightingale, but at what cost? An auto-ethnography of a volunteer frontline mental health trainer’s experiences during the COVID-19 pandemic
Source: J Health Psychol. 2023 Dec 11;29(6):534–51. doi: 10.1177/13591053231213478 (PMC11075404; doi:10.1177/13591053231213478)
Supplement: sj-docx-2-hpq-10.1177_13591053231213478 – Supplemental material for Responding to the call of the NHS Nightingale, but at what cost? An auto-ethnography of a volunteer frontline mental health trainer’s experiences during the COVID-19 pandemic [file sj-docx-2-hpq-10.1177_13591053231213478.docx]

Syntax

As this paper reports a qualitative, auto-ethnographic, analysis, syntax of the type used in statistical software is not relevant. As suggested in the author guidance, instead, the analysis methods used can be provided. As noted in the main text:

“Autoethnography represents the qualitative paradigms of constructivism and interpretivism, which together suggest that the notion of reality and knowledge is socially constructed, context-specific and individually interpreted. It is because of this theoretical standpoint that autoethnographic enquiry picks up on the highly nuanced and complex nature of what it is to experience something and what it is to be impacted by something – both these phenomena are highly sensitive to individual context. Alternative methods are not able to achieve this to such a degree as they rely on empirical evidence, struggle methodologically to capture context, and adopt a positivist approach, which seeks to find one independent reality.

[…]

After a two-week period of deliberately distancing myself from the data, I transcribed the voice message data verbatim and added it to my fieldnotes. I then exported both sets of data into NVivo (Pro V.12) and reviewed it as a chronological narrative timeline. Reflexive thematic analysis, as outlined by Braun & Clarke (2020), was chosen because of its shared epistemological and ontological orientation with ethnography. It upholds the subjective experience as an analytical and interpretive resource by which to produce insight and theory. In this way, the analytical process began through total engagement with the data. Broad coding was used to identify patterns within the data of interesting sociocultural experiences and behaviours. Similar codes were then clustered and thematic development occurred inductively throughout a process of auto-enquiry and reflexive dialogue. Memos (in NVivo) were used to document the reflexive process, capturing the maturation of themes and their interpretation within the social context of the Nightingale. The final themes were chosen as per their significance to the research question, and visually presented to illustrate their interactions and meaning”
