# Supplementary material for: Responding to the call of the NHS Nightingale, but at what cost? An auto-ethnography of a volunteer frontline mental health trainer’s experiences during the COVID-19 pandemic
Source: J Health Psychol. 2023 Dec 11;29(6):534–51. doi: 10.1177/13591053231213478 (PMC11075404; doi:10.1177/13591053231213478)
Supplement: sj-docx-3-hpq-10.1177_13591053231213478 – Supplemental material for Responding to the call of the NHS Nightingale, but at what cost? An auto-ethnography of a volunteer frontline mental health trainer’s experiences during the COVID-19 pandemic [file sj-docx-3-hpq-10.1177_13591053231213478.docx]

Log file

As the software used for qualitative analysis (NVivo) does not produce log files, in line with the author guidelines we provide below the theme structure identified.

***
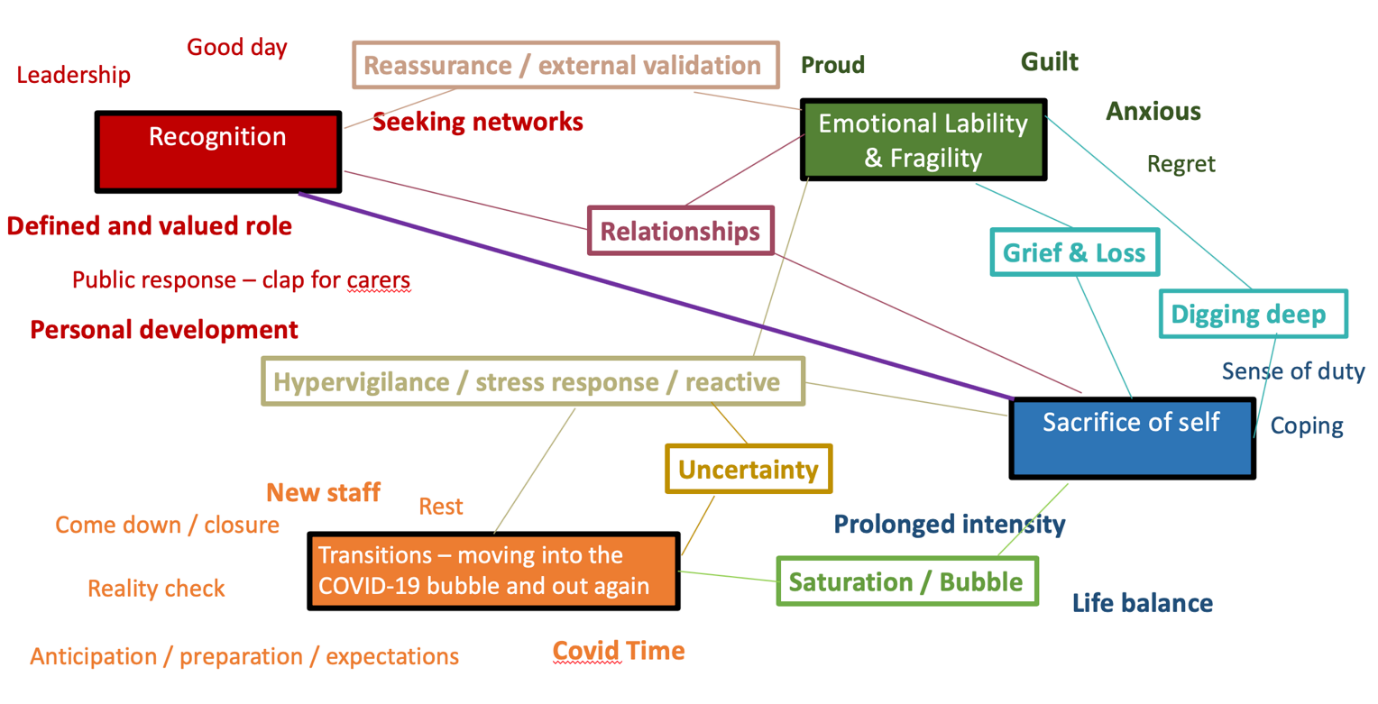
***
