# Supplementary material for: Responding to the call of the NHS Nightingale, but at what cost? An auto-ethnography of a volunteer frontline mental health trainer’s experiences during the COVID-19 pandemic
Source: J Health Psychol. 2023 Dec 11;29(6):534–51. doi: 10.1177/13591053231213478 (PMC11075404; doi:10.1177/13591053231213478)
Supplement: sj-docx-4-hpq-10.1177_13591053231213478 – Supplemental material for Responding to the call of the NHS Nightingale, but at what cost? An auto-ethnography of a volunteer frontline mental health trainer’s experiences during the COVID-19 pandemic [file sj-docx-4-hpq-10.1177_13591053231213478.docx]

Memo

The other documents provided outline the following:

Dataset: the types of data used in the analysis, and an explanation of why the raw data is not available.

Syntax: detail about the type of qualitative analysis used, as qualitative software does not produce syntax files of the type used by statistical software.

Log file: the results of the analysis, in this case, the themes identified.
